# Global Warming, Fertility, and Spermatogenesis Decline: Global and Regional Evidence from 195 Countries and Implications for Climate Adaptation Policy

**DOI:** 10.3390/ijerph23030331

**Published:** 2026-03-06

**Authors:** Ali Amini, Babak Behnam

**Affiliations:** 1 School of Public Affairs, American University, Washington, DC 20016, USA; 2 Avicenna Biotech Research, Clarksburg, MD 20871, USA; 3 Floret Advanced Genomics & Bioinformatics Research Center, Lagos 100282, Nigeria

**Keywords:** male infertility, climate change, spermatogenesis, heat stress, oxidative damage, sperm quality, environmental exposure, urban heat island, reproductive health, temperature threshold

## Abstract

**Highlights:**

**Public health relevance—How does this work relate to a public health issue?**

- Rising global temperatures represent an emerging environmental stressor with measurable associations with fertility decline across 195 countries from 1960 to 2023, with potential implications for reproductive and population health worldwide.
- The biological sensitivity of spermatogenesis to heat exposure—operating through oxidative stress, germ cell apoptosis, and disrupted chromatin integrity—provides a plausible mechanistic pathway linking climate change to declining male reproductive health at the population level.

**Public health significance—Why is this work of significance to public health?**

- This study demonstrates that the association between global warming and fertility decline is not uniform but is critically conditioned by adaptive capacity: temperature anomalies remain significant predictors of fertility decline in Africa and the Middle East even after controlling for GDP per capita, while the effect attenuates in higher-income regions such as Europe, East Asia, and the Arctic.
- These findings highlight that populations with limited access to cooling infrastructure, occupational heat protections, and robust healthcare systems may face a disproportionate reproductive burden from rising temperatures, contributing to widening global health inequities.

**Public health implications—What are the key implications or messages for practitioners, policy makers and/or researchers in public health?**

- Policymakers in low- and middle-income countries (LMIC) should integrate heat resilience strategies—including cooling infrastructure, occupational protections, and reproductive healthcare access—into climate adaptation planning to reduce climate-related reproductive vulnerability.
- Researchers should prioritize individual-level longitudinal studies linking personal heat exposure to biological reproductive endpoints (e.g., semen quality, pregnancy outcomes) to move beyond ecological associations and establish causal pathways informing evidence-based public health interventions.

**Abstract:**

This study investigates whether long-term global warming is associated with fertility decline across 195 countries from 1960 to 2023, and whether this relationship varies by economic development and adaptive capacity. We analyze Total Fertility Rate (TFR) data from the World Bank alongside temperature anomaly measures from NOAA and NASA using Pearson correlations and ordinary least squares (OLS) regression models. Regional analyses include Africa, Asia, Europe, the Middle East, and the Arctic, with GDP per capita serving as a proxy for economic development and adaptive capacity. Globally, temperature anomalies and fertility exhibit a strong negative correlation (r≈−0.90, p<0.001). However, substantial regional heterogeneity emerges after controlling for GDP. In Africa (r=−0.89) and the Middle East, temperature anomalies remain statistically significant predictors of fertility decline even after GDP adjustment (β=−0.99, p<0.001; β=−1.27, p<0.001, respectively). In contrast, temperature effects become statistically insignificant in South Asia, East Asia, Europe, and the Arctic once GDP is controlled, indicating that fertility decline in these regions is driven primarily by socioeconomic modernization rather than climatic stress. These findings suggest that global warming functions as a conditional demographic stressor whose impact depends critically on adaptive capacity. In regions with limited infrastructure, including constrained access to air conditioning, healthcare, and occupational heat protection, rising temperatures remain significant predictors of fertility decline, potentially mediated through heat-sensitive biological mechanisms such as impaired spermatogenesis. By contrast, in higher-income regions, high adaptive capacity appears to buffer reproductive systems from thermal stress, allowing socioeconomic factors to dominate fertility dynamics.

## 1. Introduction

Infertility affects approximately 15% of couples worldwide, and male factors are implicated in approximately 40–50% of cases alone or in conjunction with female factors [1,2,3]. In this article, we propose that climate-induced impairment of male reproductive function represents a critical biologically mediated pathway through which heat stress may influence demographic and socioeconomic trends. For much of the twentieth century, male infertility remained under-recognized due to limited diagnostic tools, social stigma, and a predominant focus on female reproductive health. Over the past two decades, however, advances in semen analysis, molecular diagnostics, proteomics, and large-scale epidemiological studies have reframed infertility as a shared reproductive burden, with male contributions now understood to play a central and quantifiable role in reproductive outcomes [3].

Currently, the human environment has undergone profound changes. Climate records demonstrate a marked rise in the frequency, duration, and intensity of extreme heat events globally, including an increasing number of days with ambient temperatures surpassing 33 °C, near or above the scrotal thermoregulatory threshold, across equatorial, subtropical, and densely urbanized regions [4,5]. The urban Heat Islands further amplify these peaks. The burden of heat exposure is now quantifiable not only in calendar days but in population-weighted “person-days,” which have surged sharply in the twenty-first century.

This climatological shift aligns with emerging clinical evidence suggesting that ambient heat stress is an underappreciated contributor to declining semen quality. A multi-center Chinese cohort of 33,234 men showed significant negative associations between heat anomalies during hot seasons and key semen parameters, sperm concentration, total motile sperm count, and progressive motility [6]. Complementary physiological studies indicate that heat stress affects spermatogenesis through multiple mechanisms: disruption of the hormonal axes, destabilization of mitochondrial function, increased reactive oxygen species (ROS) and defects in the sperm chromatin compaction essential for fertility [7].

It is worth noting that heat stress does not act in isolation. Semen is uniquely receptive to environmental pollutants, such as PFAS, which can bind to protamines and perturb chromatin compaction [8,9]. Temperature increase may exacerbate these effects. Heat accelerates oxidative damage, pollutant-bound protamines more susceptible to fragmentation [10,11].

This synergistic susceptibility provides a mechanistic explanation for observed seasonal declines in sperm quality, where semen parameters worsen in hotter months when both heat exposure and pollutant burdens tend to rise [8]. This micro-level evidence establishes a plausible biological chain linking rising temperatures to impaired spermatogenesis and increased population-level risk of subfertility or infertility.

This article synthesizes this micro-level evidence with macro-level climate and demographic data to test the hypothesis that global climate change may contribute to impaired spermatogenesis and, through this biological pathway, influence fertility trends.

It should be noted that this study employs an ecological design at the country-level. While we report population-level associations between temperature change and fertility, such patterns cannot be interpreted as direct evidence of individual-level reproductive impairment or confirmation of specific biological mechanisms. The micro-level thermophysiological pathways discussed above are presented as biologically plausible and experimentally supported mechanisms that may help contextualize observed demographic trends, but they are not directly tested in the country–year analyses conducted here. National fertility rates reflect complex social, economic, cultural, and institutional processes that extend far beyond individual sperm parameters. Accordingly, our empirical analysis focuses on large-scale co-movement between climate and fertility outcomes rather than causal inference regarding biological impairment.

While the social science literature has long established that population-level fertility decline is driven primarily by socioeconomic development [12,13,14], recent biomedical research demonstrates a causal relationship between heat exposure and reproductive impairment [6,15]. These findings suggest that ambient temperature operates as a biological constraint on reproduction that exists alongside, rather than in place of, social and economic forces.

These findings suggest that ambient temperature may operate as a biological constraint on reproduction that interacts with social and economic forces. In this study, we examine the long-run correlation between ambient temperature change and fertility using country–year data from 195 countries spanning 1960 to 2023. We find a very strong bivariate association between rising temperatures and declining births per woman. When we estimate regression models that account for economic development using GDP (Gross Domestic Product) per capita, this relationship becomes highly heterogeneous across regions. In Africa and the Middle East, temperature anomalies remain strong and statistically significant predictors of fertility declines even after controlling for income, whereas in high-income regions the temperature effect becomes statistically insignificant once GDP is included.

This pattern is consistent with a model in which heat acts as a conditional demographic stressor. In low-and middle-income countries (LMIC) with limited adaptive capacity, temperature anomalies remain statistically associated with fertility decline, suggesting that environmental stress may interact with structural vulnerabilities. In higher-income regions, and adaptive capacity appear to attenuate this association, allowing socioeconomic factors to dominate fertility dynamics. Rather than demonstrating direct biological causation, these findings highlight how environmental and developmental contexts may jointly shape fertility outcomes at the population level.

These findings illustrate a framework in which climate warming may interact with the demographic transition in ways that vary by socioeconomic context. Rather than identifying temperature as a universal driver of fertility decline, the study characterizes climate warming as a conditional demographic correlate whose association with fertility depends critically on adaptive capacity, thereby encouraging further interdisciplinary research into how biological vulnerability, institutional resilience, and environmental stress intersect over the long run. Throughout this manuscript, we use the term adaptive capacity to refer to the economic, institutional, infrastructural, and healthcare resources—proxied empirically by GDP per capita [16,17,18]—that enable populations to buffer environmental stress through access to climate-controlled housing, occupational heat protections, medical services, and public health systems. This usage aligns with the IPCC definition of adaptive capacity as the ability of systems, institutions, and populations to adjust to climate variability and moderate potential damages [19], and reflects broader conceptualizations emphasizing governance quality, social organization, and institutional effectiveness in shaping societal responses to climate stressors [20,21].

## 2. Thermoregulatory Physiology of the Scrotum: Heat Exposure and Male Fertility

### 2.1. Thermoregulatory Physiology of the Scrotum and Male Fertility

**Figure 1:**
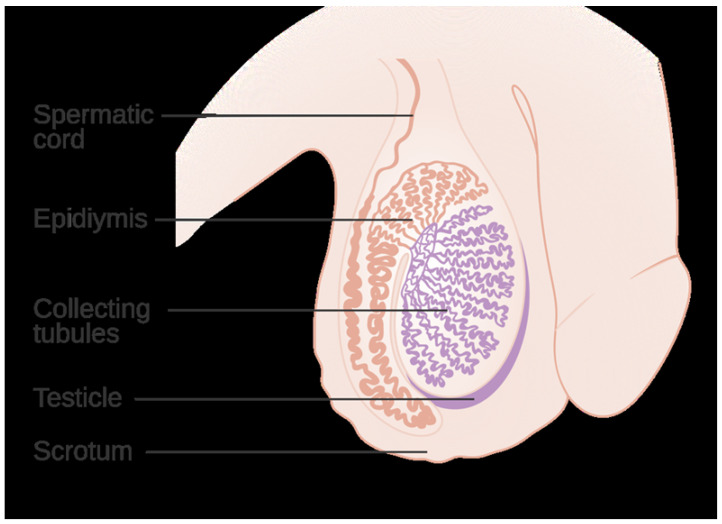
Anatomy of the human scrotum and testes, showing the testes in the scrotum connected via the spermatic (spermatic) cord. Key thermoregulatory elements are labeled (pampiniform plexus around the testicular artery, dartos muscle in scrotal skin, etc.). These structures maintain testicular temperature ∼2–4 °C below core body temperature for optimal spermatogenesis. The testes are housed outside the body to keep them cooler. The pampiniform plexus (venous network in the spermatic cord) counter-current cools arterial blood entering the testis. The tunica dartos (smooth muscle in the scrotal wall) and the cremaster muscle (skeletal muscle in the cord) contract or relax to adjust scrotal surface area and testis position, drawing the testis closer to the body when cold and lowering them when warm. Together with the thin, lightly insulated scrotal wall and scrotal vasomotor responses (sweating and vasodilation), these mechanisms hold the testis at the ideal ∼34 °C (about 3–4 °C below core) required for normal sperm production. (Image [45]).

The anatomical placement of the testes within the scrotum (See Figure 1), away from the abdominal cavity, is a fundamental biological adaptation that maintains testicular temperature roughly 2–4 °C below core body temperature (~37 °C), a prerequisite for optimal spermatogenesis [22]. In humans, scrotal thermoregulation relies on several active and passive mechanisms, including the cremaster and dartos muscles adjusting testicular position, a counter-current vascular heat exchange in the spermatic cord, minimal insulating subcutaneous fat, and scrotal sweating or vasodilation of superficial blood vessels in warm conditions [23,24]. Spermatogenesis is most efficient at testicular temperatures of approximately 34–35 °C (a few degrees below core temperature). Elevation of scrotal temperature, even modestly, disrupts this finely tuned environment and impairs reproductive outcomes [25,26]. Sustained heat exposure causes increased spermatogenic cell apoptosis and reduced germ cell survival, especially during the sensitive meiotic and spermiogenic phases [4]. This is accompanied by disruption of Sertoli cell function and the blood–testis barrier, leading to impaired support of developing germ cells and reduced spermatid maturation [22].

Second, heat stress induces oxidative stress and mitochondrial dysfunction in spermatozoa, with decreased ATP (Adenosine Triphosphate) production, reduced membrane potential, and impaired motility [26]. These cellular disturbances also produce abnormal sperm morphology and structural defects, often attributable to disrupted cytoskeletal formation, heat-induced disruption of protein folding, and altered gene expression during spermiogenesis [26,27]. Additionally, elevated temperatures increase sperm DNA fragmentation and chromosomal abnormalities, stemming from meiotic failure, impaired recombination, and reduced efficacy of DNA repair processes [26,28]. Animal models further underscore this vulnerability. High ambient temperature in seasonal heat-stress studies (e.g., Angora rabbits) induces massive transcriptomic changes in testicular tissue, morphological damage to seminiferous tubules, and prolonged recovery periods for normal spermatogenesis [29].

Testicular heat stress interventions in rodent models show that recovery of normal germ cell cycles may take multiple months, and repeated or chronic heat exposure can lead to persistent fertility impairment. In summary, the physiology indicates that prolonged exposure to ambient heat above the scrotal cooling threshold is not merely a nuisance but a potent, direct stressor on male fertility [30].

Importantly, many of the microenvironmental vulnerabilities described above for the testis are mirrored in female reproductive tissues and the maternal–fetal interface. Heat and other environmental stressors (pollutants, particulate matter, and metabolic insults) provoke reactive oxygen species (ROS) accumulation, mitochondrial dysfunction, and disrupted cell–cell/ECM interactions in the ovary, endometrium, cervix, and placenta: mechanisms that converge on impaired gametogenesis, implantation failure, placental insufficiency, and adverse obstetric outcomes. Recent systematic and bibliometric reviews of placental oxidative stress and reproductive oxidative stress highlight how ROS driven signaling and redox imbalance are central pathways linking environmental exposures to adverse pregnancy and reproductive outcomes [31,32].

### 2.2. Thermoregulatory Mechanisms and Spermatogenic Sensitivity

Thermoregulatory cooling mechanisms (muscular adjustment of testis position, vascular heat exchange, minimal insulation, scrotal sweating/vasodilation) are critical. When ambient heat exceeds the scrotal threshold (about 33–34 °C), these mechanisms can be overwhelmed, and adverse outcomes ensue. Key mechanisms include:

#### 2.2.1. Germ-Cell Apoptosis and Barrier Disruption

Sustained scrotal heat causes apoptosis in spermatocytes and spermatids and disrupts Sertoli cell support and the blood–testis barrier [30]. Parallels in female tissues are apparent: in the placenta and decidua, excessive oxidative burden can dysregulate autophagy and apoptosis in trophoblasts, impairing invasion and spiral artery remodeling and contributing to clinical syndromes such as preeclampsia and fetal growth restriction. Decidualization has evolved mechanisms to tolerate physiological oxidative flux, but when overwhelmed these pathways fail and cell death ensues (recent reviews detail decidual resistance mechanisms and failure under stress) [33].

#### 2.2.2. Oxidative Stress and Mitochondrial Dysfunction

Heat stress elevates ROS in sperm, damaging mitochondria and reducing motility and viability [26]. Analogously, excessive ROS in the endometrium and placenta impairs mitochondrial function, diminishes antioxidant defenses, and perturbs angiogenic signalling (HIF/VEGF), compromising tissue oxygenation and nutrient exchange. Systematic reviews of oxidative stress biomarkers in pregnancy document associations between elevated OS markers and adverse pregnancy outcomes, underscoring the clinical relevance of redox imbalance across reproductive tissues [32].

#### 2.2.3. ECM Remodeling and Chromatin Integrity

Elevated temperature increases sperm DNA damage and abnormal chromatin compact [30]. In female reproductive sites, ROS and inflammation activate matrix metalloproteinases (MMPs) and NF κB/p38 signalling that remodel the extracellular matrix (ECM), as a necessary process in ripening and repair but one that, if dysregulated by environmental stress, leads to pathological ECM breakdown, scarring, or poor wound healing (for example, in the cervix or postpartum perineum). These ECM pathways are functionally linked to redox state and local metabolic cues in the microenvironment [34,35].

#### 2.2.4. Long Term Impacts and Recovery Dynamics

Chronic or repeated heat exposures in animal models lead to prolonged recovery of spermatogenesis and persistent functional impairment [29,30]. Female reproductive tissues also show protracted recovery patterns after environmental insult: placental or endometrial injury can have downstream impacts on subsequent cycles and pregnancy outcomes, and impaired wound healing in the perineum or endometrium can be modulated by local metabolites (see lactic acid discussion below).

#### 2.2.5. Clinical/Wound Healing Insight

Recent systematic evidence shows that lactic acid topical therapy accelerates episiotomy wound healing, reduces infection rates, and modulates local pH and angiogenic responses [31]. These findings illustrate an important mechanistic principle: microenvironmental metabolites (e.g., lactate) and pH influence cell migration, fibroblast activity, angiogenesis, and innate immune responses, processes central to both perineal wound repair and to regenerative events in the endometrium and cervical epithelium. Therefore, metabolic shifts induced by heat or pollutant-driven tissue hypoxia that alter lactate production or local acidity could plausibly impair repair and remodeling in multiple reproductive tissues.

### 2.3. Molecular Mechanisms: Temperature-Sensitive Reproductive Proteins

**Figure 2:**
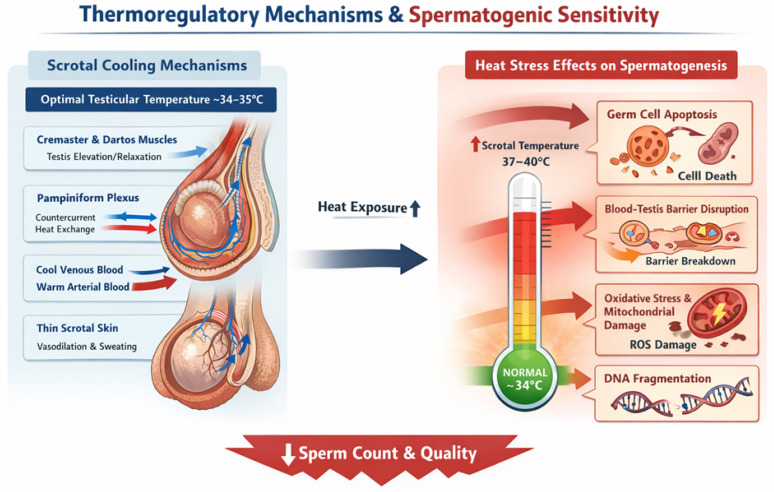
The testes are positioned within the scrotum to maintain a temperature approximately 2–4 °C below core body temperature (∼37 °C), with optimal spermatogenesis occurring at ∼34–35 °C. Scrotal thermoregulation relies on coordinated mechanisms including cremaster and dartos muscle–mediated positional adjustment, counter-current heat exchange in the pampiniform plexus, minimal subcutaneous insulation, and scrotal vasodilation and sweating. When ambient heat exceeds the scrotal cooling threshold (∼33–34 °C), testicular temperature rises toward core levels, overwhelming these protective systems. Elevated temperature induces germ cell apoptosis, disrupts Sertoli cell support and the blood–testis barrier, increases oxidative stress and mitochondrial dysfunction, and promotes DNA fragmentation. These effects collectively reduce sperm count, motility, morphology, and genomic integrity, illustrating the narrow thermal window required for normal spermatogenesis. (Image created by AI (GPT 5) under supervision of authors).

Several proteins in the testis and sperm function as precise temperature sensors and effectors (Table 1) [34,35,36]. For example, the CatSper Ca^2+^ channel remains closed until ∼33.5 °C, then opens to trigger sperm hyperactivation [36,37,38]. Heat also alters germ cell transcription: key metabolic and antioxidant genes (Acly, Txnrd1, etc.) are downregulated in spermatocytes exposed to elevated temperatures, reflecting active germline reprogramming [30,39]. Mild stress upregulates heat-shock proteins (HSP70/90) and piRNA pathway components to stabilize proteins and protect the germline; but when stress is prolonged, apoptotic factors (BAX, caspases) dominate, culling damaged cells [30,39]. A polyamine in seminal plasma, spermine, reversibly inhibits this temperature gating, preventing premature activation before sperm enter the female reproductive tract [35]. In parallel, TRPV4 has been identified as a temperature-activated depolarizing channel in human sperm that initiates the electrical and calcium signaling cascade leading to CatSper activation [35] (See Figure 2).

**Table 1:** Key sperm and germline proteins affected by temperature stress.

Protein/Pathway	Primary Function	Temperature Sensitivity	Cell Type/Expression
**CatSper** [36,37,38]	Ca^2+^ channel essential for hyperactivated sperm motility and chemotaxis	Directly temperature-gated; activates above 33.5 °C	Sperm flagellum (mature spermatozoa)
**TRPV4**(DSper) [35]	Cation channel initiating membrane depolarization and Ca ^2+^ influx	Activated by warm temperatures (approximately ≥34 °C)	Sperm plasma membrane (primarily human)
**Metabolic enzymes (Acly, SelV, SLC16A7, Txnrd1, Prkar2B)** [34]	Energy metabolism, redox regulation, lactate transport, and signaling	Transcriptionally downregulated by at least two-fold under heat stress	Pachytene spermatocytes, round spermatids
**Heat shock proteins (HSP70, HSP90)** [39]	Molecular chaperones preserving protein folding and stability	Strongly upregulated as a protective heat stress response	All germ cell types, especially spermatocytes and spermatids
**Components of piRNA Biogenesis Machinery** [39]	Silencing of transposable elements and maintenance of genome integrity	Upregulated during heat stress in coordination with heat shock response	Pachytene spermatocytes, round spermatids
**Apoptotic proteins (BAX, caspase-3, caspase-9)** [30]	Programmed elimination of thermally damaged germ cells	Activated under prolonged or severe heat exposure above 35 °C	Spermatocytes and early spermatids

Note: Temperature thresholds refer to scrotal or testicular temperature, typically 2–4 °C below core body temperature. CatSper = cation channel of sperm; TRPV4 = transient receptor potential vanilloid 4; Acly = ATP citrate lyase; SelV = selenoprotein V; SLC16A7 = monocarboxylate transporter; Txnrd1 = thioredoxin reductase 1; Prkar2B = protein kinase A regulatory subunit.

Cross-tissue parallels and mechanistic complements—Many of these molecular stress responses extend to non-testicular reproductive tissues. Heat-shock proteins and antioxidant enzymes are induced in oocytes, granulosa cells, trophoblasts and decidual cells under thermal or oxidative challenge, functioning to stabilize proteomes and protect key developmental programs; but when stress is prolonged, apoptotic and senescence pathways dominate, compromising tissue function. HIF-1α mediated hypoxic signalling and downstream VEGF angiogenic responses, central to placental vascular development, are sensitive to ROS and pollutant exposures; dysregulation produces anti-angiogenic shifts (e.g., sFlt-1 elevation) that impair the fetomaternal interface. Reviews of placental pathogenesis and reproductive toxicology highlight PFAS, microplastics, and metal exposures as drivers of ROS production and perturbation of HIF/VEGF and NF-κB pathways in the placenta and ovary, mirroring mechanisms implicated in testicular heat injury [31,40].

#### 2.3.1. Cryptorchidism

In undescended testes, exposure to abdominal temperatures near 37 °C disrupts heat- 168 sensitive transcriptional programs in spermatocytes and spermatids and can aberrantly 169 activate CatSper channels, contributing directly to impaired fertility.

#### 2.3.2. Evolution of Testicular Cooling

The discovery that CatSper is sharply gated above 33.5 °C provides a compelling molecular rationale for the evolution of extra-abdominal testes in mammals. By maintaining sperm below this activation threshold, testicular cooling prevents premature motility and preserves sperm function until they encounter the appropriate physiological environment in the female reproductive tract.

TSGA10 (Testis Specific Gene 10) is a major sperm protein recently proposed as a thermoregulator. It is cleaved into two fragments: one supports sperm tail structure and energy supply, while the other stabilizes mitochondrial energy production. By interacting with cytochrome c1 (CytC1), it prevents harmful heat and reactive oxygen species (ROS) generation. During thermal stress, this function fails, leading to ROS buildup and cell damage. Its additional role in sequestering HIF1α suggests it integrates temperature, oxygen, and survival signals, positioning it as a likely functional protein responding to temperature, potentially via apoptosis-related pathways [41,42,43].

In summary, the testis and other reproductive tissues share a conserved stress-response architecture:

- A metabolic/redox node (mitochondrial ROS generation, antioxidant enzymes such as SOD/Txnrd, and NAD^+^/NADH balance),
- A chaperone/protein quality node (HSPs, unfolded protein response),
- A structural/ECM node (MMPs, collagen crosslinking, matricellular signaling): environmental stressors shift tissues from adaptive responses (chaperone induction, controlled ECM remodeling) toward maladaptive outcomes (oxidative damage, apoptosis, excessive ECM degradation, or fibrosis), and these shifts explain much of the cross-tissue vulnerability in reproductive biology.

### 2.4. Environmental Stress and Other Reproductive Tissues

Environmental stressors (heat, pollutants, PFAS/microplastics, and metabolic insults) similarly perturb microenvironments across the female reproductive tract:

- Placenta: Exposures elevate ROS, dysregulate HIF-VEGF signalling, and increase anti-angiogenic mediators, leading to impaired trophoblast invasion, abnormal vascular remodeling, and increased risk of preeclampsia, fetal growth restriction and preterm birth. Reviews emphasize placental oxidative stress as a central mediator linking environmental insults to pregnancy complications [31,32].
- Ovary & folliculogenesis: Heat and pollutants impair steroidogenesis (reduced StAR, aromatase), increase granulosa-cell apoptosis, alter follicular fluid composition (oxidative markers), and reduce oocyte quality, mechanistic pathways observed in mammalian models and human observational studies. Microplastic and PFAS literature ties reproductive-toxic outcomes to oxidative stress and endocrine disruption [44].
- Endometrium, cervix, wound repair: Local redox balance and metabolite signals (notably lactate) modulate immune cell recruitment, angiogenesis, fibroblast activation, and MMP activity required for receptivity and repair. Brezeanu et al. showed that lactic-acid–based topical therapy speeds episiotomy healing and reduces infection, an observation that supports the concept that metabolite/pH modulation of the microenvironment meaningfully alters repair trajectories in reproductive tissues [31].

Thus, while the testes are uniquely thermally exposed by virtue of scrotal anatomy, the microenvironmental principles that govern injury and repair (redox homeostasis, chaperone activation, ECM dynamics, angiogenesis and metabolite signaling) are shared across reproductive tissues and environmental stressors exploit these same conserved nodes to produce dysfunction in ovaries, uterus, placenta, cervix, and repair processes.

### 2.5. Climate Change and Biological Systems

Rising global temperatures have demonstrated profound effects on biological systems across multiple scales. From coral bleaching events to shifts in species migration patterns, temperature sensitivity appears to be a fundamental constraint on biological function. Recent work on the analysis and interpretation of critical temperatures demonstrates that organisms exhibit temperature thresholds, quantified as critical thermal maxima (CT*_max_*) and minima, beyond which failure rates sharply increase, and essential biological processes begin to collapse. Male fertility, in particular, has long been recognized as temperature-sensitive. The evolution of external testes in mammals reflects this sensitivity, maintaining sperm production at temperatures 2–3 °C below core body temperature. However, the molecular mechanisms underlying this sensitivity, particularly the role of specific proteins like TSGA10, remain poorly understood.

### 2.6. Impact of Temperature on Fertility

**Figure 3:**
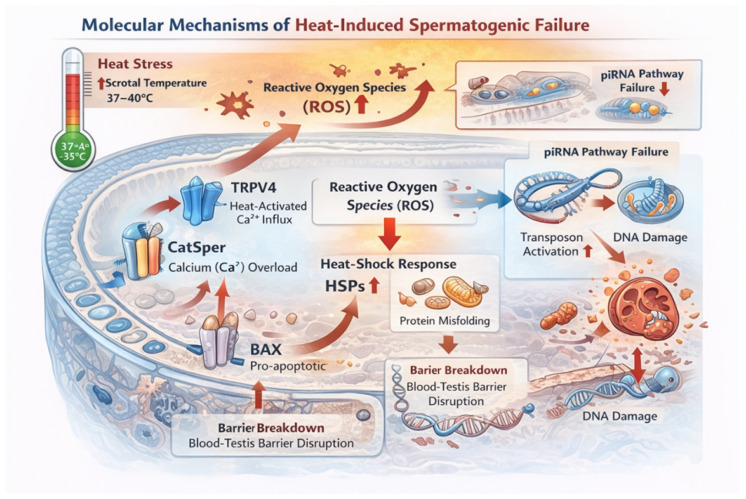
Heat stress elevates testicular temperature and activates temperature-sensitive ion channels, including TRPV4 and CatSper, leading to calcium influx and altered sperm signaling. Increased reactive oxygen species (ROS) production disrupts mitochondrial function and redox balance. Mild stress induces protective heat-shock proteins (HSP70/90) and piRNA pathway components to stabilize protein folding and preserve genome integrity. However, sustained or severe heat exposure suppresses metabolic and antioxidant gene expression, impairs piRNA-mediated transposon silencing, and activates pro-apoptotic mediators such as BAX and caspases. Concurrent disruption of the blood–testis barrier further compromises germ cell survival. These interconnected molecular pathways converge on apoptosis, chromatin damage, and impaired spermatogenic progression, linking thermosensitive signaling to functional sperm decline.(Image created by AI (GPT 5) under supervision of authors.

Heat exposure impairs reproductive health primarily through biological disruption, especially male spermatogenesis, rather than reduced sexual activity [46]. Proteins like TSGA10, which are highly expressed in sperm, play dual roles in structuring mitochondria for ATP generation and contributing to thermogenesis. Elevated temperatures destabilize such proteins, compromising sperm energy metabolism and motility, thus reducing fertility (Figure 3). Empirical evidence supports these mechanisms globally. Ref. [47] found that heat exposure lowers conception rates in Hungary, forecasting a 1% annual decline by 2050 due to climate-driven pregnancy loss. In South Korea, ref. [48] reported reduced births nine months after heatwaves, confirming a biological rather than socioeconomic effect. Similarly, [49] showed that extreme heat in the U.S. shortens pregnancies, causing over 150,000 lost gestational days annually, disproportionately affecting Black mothers. A global meta-analysis of 198 studies [50] confirmed that each 1 °C rise increases preterm birth risk by 4%, with heatwaves raising it by 26%, alongside heightened risks for stillbirth and congenital anomalies.

Cold exposure can also threaten pregnancy outcomes. Ref. [51] found that cold spells in China increased preterm births by up to 18%, particularly late-stage spontaneous cases. Earlier, ref. [47] identified a 0.52% rise in early embryonic loss linked to post-conception heat exposure in 6.5 million Hungarian pregnancies, the first causal evidence that heat elevates embryo mortality.

At the individual level, ref. [52] found that cumulative male heat exposures (e.g., hot baths, fever) reduced fecundability, especially in men over 30. From a global perspective, ref. [15] reported a U-shaped relationship between ambient temperature and female infertility across 174 countries, with lowest risk near 15 °C; both heat and cold deviations elevated infertility rates.

Mechanistically, heat stress induces oxidative damage, microbial imbalance, and disrupted testicular metabolism [53], impairing spermatogenesis and increasing DNA fragmentation [10,11]. For females, heat compromises ovarian function, oocyte quality, placental blood flow, and fetal development [10,54], consistent with findings in animal models where heat stress drastically lowers fertility [55].

Beyond biology, refs. [56,57] highlight socioeconomic amplifiers—climate-induced fertility reductions in industrialized nations and divergent demographic pressures in LMIC. As [58] emphasize, reproductive decline at sublethal temperatures may be the most immediate threat climate change poses to biodiversity and human population stability.

Diverse evidence from physiological, epidemiological, and ecological data converges on a clear conclusion: rising global temperatures pose a pervasive threat to reproductive health, operating through oxidative, endocrine, and developmental pathways that span from sperm function to fetal survival. Climate change thus represents not only an environmental and public health emergency but also a profound reproductive crisis with lasting demographic and evolutionary implications.

### 2.7. Climate Change and the Expansion of Extreme Heat

In the 1950s the world population was roughly 2.5–2.6 billion, and days with very high ambient temperatures (e.g., >33 °C) were largely confined to deserts, some equatorial savannas, and brief seasonal extremes; most temperate and humid tropical rainforest zones experienced relatively few prolonged episodes above that threshold [59]. World Population Prospects Crucially, instrumental and reanalysis records show that the frequency, intensity and seasonal length of hot extremes have increased since the mid-20th century, with heatwave days and extreme-temperature events becoming substantially more common across most land areas which is a trend that is robustly documented in the Intergovernmental Panel on Climate Change (IPCC) assessment [60].

By the 2010s–2020s, the global population had grown to about 8 billion, concentrating many more people in regions that are already climate hotspots. Population growth, urbanization and the rapid expansion of cities amplify exposure: city populations are larger and often experience local warming above regional background (the Urban Heat Island effect), which observational and review studies show can increase urban temperatures by several degrees relative to nearby rural areas (typical nighttime UHI intensities of a few °C and local peaks that exceed +3–6 °C in some cases). These urban increments act on top of the background climate warming and materially increase the number of days and nights when ambient air temperatures exceed physiologically important thresholds [61].

The combination of climate warming and population/geographic concentration yields far larger population-weighted exposures than changes in raw climate alone. For example, a global study that combined high-resolution daily temperature fields with longitudinal urban population data found urban person-days of exposure to extreme heat rose from 40 billion in 1983 to 119 billion in 2016 (a 199% increase), with most of the increase driven by both warming and urban population growth. This “person-days” metric makes clear that even moderate increases in the frequency of hot days can translate into very large increases in human heat exposure [62].

Regionally, the effects are already extreme. South Asia, parts of the Middle East and North Africa, and some equatorial and subtropical African regions now routinely experience dozens to many hundreds of days per year at very high temperatures; analyses and public-health assessments (including UNICEF and climate-attribution studies) report that large cohorts, hundreds of millions, already face many weeks to months per year of conditions above labor- and health-risk thresholds (e.g., 30–35 °C daytime or wet-bulb thresholds in humid zones), and attribution work shows human-caused warming has made recent deadly heat events much more likely and intense.

Taken together, the mid-20th-century baseline (low population, fewer hot-day exposures) and the present (≈8 billion people, expanded urban areas, and a climate with far more frequent, longer, and more intense hot spells) create a situation where person-days above physiological thresholds (such as ∼33 °C) have increased several-fold. That multi-fold increase in population heat exposure is the proximate environmental change underlying hypotheses that chronic or repeated ambient heat stress could drive population-level impacts on heat-sensitive biological systems (including spermatogenesis) [63].

**Figure 4:**
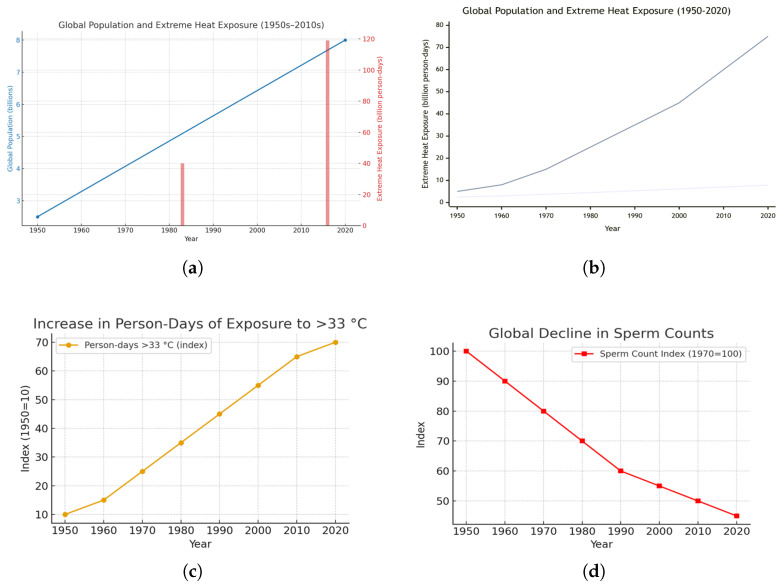
Global Population Growth, Extreme Heat Exposure, and Decline in Sperm Counts (1950–2020). (**a**) Global population growth (UN DESA, 2022 [64]) and the expansion of annual days exceeding 33 °C, adapted from IPCC (2021) [65] and NOAA NCEI (2023) [66] datasets. The population curve is smoothed to illustrate the long-term trend. (**b**) Global increase in combined population and extreme heat exposure (billion person-days) from 1950 to 2020, showing the acceleration of thermal exposure with climate warming [67]. (**c**) Indexed rise in person-days of exposure to ambient air temperatures above 33 °C (1950s–2020s), demonstrating more than a six-fold increase over the study period. (**d**) Global decline in sperm counts (1970s–2020s) indexed to 1970 = 100, based on meta-analytic evidence showing consistent decreases across continents. Together, these panels highlight how climate change has dramatically intensified population exposure to extreme heat, with exposure increasing far faster than population growth, and how thermal stress coincides with global declines in male reproductive health. Data sources: IPCC (2021) [65]; NOAA NCEI (2023) [66]; UN DESA (2022) [64]; and Mukherjee et al. (2021) [67].

Figure 4 illustrates global population growth and the concurrent rise in population-weighted exposure to extreme heat, expressed as billions of person-days exceeding 33 °C. Panel (a) shows the steady increase in global population from approximately 2.5 billion in 1950 to about 7.8 billion in 2020, based on UN DESA (2022) [64] estimates. Panel (b) demonstrates that, over the same period, exposure to extreme heat has accelerated sharply—particularly since the 1980s—reflecting the combined effects of population growth, rapid urbanization, and anthropogenic climate warming [65,66,67]. Panel (c) highlights that population exposure to ambient air temperatures above 33 °C has increased more than six-fold since the 1950s, far outpacing population growth alone and underscoring that global warming is the dominant driver of expanding heat-related risk.

Panel (d) depicts the corresponding global decline in sperm counts from the 1970s to 2020s, indexed to 1970 = 100. These data are derived from a systematic review and meta-regression analysis aggregating 283 studies across 53 countries and more than 57,000 men, showing a significant and accelerating decline in sperm concentration (millions per mL), particularly after 2000 [68].

In sum, the panels illustrate a striking parallel between the intensification of global thermal exposure and the persistent decline in male reproductive health. Data sources include IPCC (2021) [65], NOAA NCEI (2023) [66], UN DESA (2022) [64], and ([67]), along with meta-analytic evidence from Levine et al. ([68]).

## 3. Data and Methods

### 3.1. Study Design

This is an ecological study using country-level aggregate data to examine associations between temperature anomalies and fertility rates across 195 countries from 1960 to 2023. The analysis employs a repeated cross-sectional design with annual observations, examining both global trends and region-specific patterns. We use correlation analysis and ordinary least squares (OLS) regression models to assess the relationship between temperature change and fertility, with and without adjustment for economic development (GDP per capita). As an ecological study, findings represent population-level associations and cannot be interpreted as evidence of individual-level causal relationships.

### 3.2. Fertility Data

We use Total Fertility Rate (TFR) data from the World Bank World Development Indicators (WDI) dataset for the period 1960–2023. The TFR is defined as the number of children a woman would bear if she experienced the age-specific fertility rates of a given year throughout her reproductive life [69]. The indicator “Fertility rate, total (births per woman)” is compiled by the United Nations Population Division (World Population Prospects 2024 Revision) from national vital registration systems, censuses, and large-scale surveys. Where vital registration data were incomplete, model-based adjustments using demographic survey data were applied to ensure reliability. Fertility data were aggregated by continent (Africa, Asia, Europe, and the Arctic) using population-weighted means to align with global coverage.

### 3.3. Temperature Data

Temperature anomaly data were sourced from NOAA’s Global Surface Temperature Analysis (NOAAGlobalTemp) [66]. We also used NASA’s GISTEMP v4 [70] as a robustness check on global trends. GISTEMP v4 blends land-based records from the Global Historical Climatology Network-Monthly and ocean-based records from the Extended Reconstructed Sea Surface Temperature. Anomalies were calculated relative to the 1951–1980 baseline, consistent with IPCC AR6 standards. Regional means for Africa, Asia, Europe, and the Arctic were extracted from NOAA’s Climate at a Glance interface, while global means were derived from NOAA and NASA combined datasets. These temperature records span 1850–present; annual means for 1960–2023 were used to align with the fertility data time frame.

### 3.4. Regional Aggregation

We analyzed both global and regional trends for four major regions: Africa, Asia, Europe, and the Arctic. Regional temperature anomalies are derived from NOAA’s area-weighted averages of gridded annual temperature anomalies, while fertility rates are aggregated using population weighting to capture each country’s relative demographic contribution. The Arctic region is defined as including countries with the majority of land area above 60° N latitude, following the Arctic Monitoring and Assessment Program (AMAP) classification.

### 3.5. Statistical Modeling

Linear regression models were used to estimate the change in fertility associated with a one-degree Celsius increase in temperature anomalies. For each region, two model specifications were estimated: Model (1) includes temperature anomalies only, and Model (2) additionally controls for log GDP per capita in order to account for economic development and adaptive capacity. Locally estimated scatterplot smoothing (LOESS) was used to visualize potential non-linear relationships between temperature and fertility. Statistical significance was evaluated at α=0.05, and 95% confidence intervals were computed for all correlation and regression estimates.

We examine the relationship between fertility and temperature anomalies using three complementary approaches: Pearson’s correlation, ordinary least squares (OLS) regression, and lagged correlation analysis. We employ these methods to capture both immediate and delayed associations between temperature changes and fertility rates. We specify linear regression models as:

$${\mathrm{Fertility}}_{t}=\beta_{0}+\beta_{1}\left(\mathrm{Temperature}\;{\mathrm{Anomaly}}_{t}\right)+\beta_{2}\left({\mathrm{GDP}}_{t}\right)+\varepsilon_{t}$$

Linear regression models estimated the slope of fertility decline per 1 °C increase in temperature anomaly, while locally estimated scatterplot smoothing (LOESS) was used to visualize potential non-linear relationships. Statistical significance was assessed at α=0.05. For each region, 95% confidence intervals were computed for correlation and regression estimates. Lagged correlations were tested at 1-, 2-, and 3-year intervals to assess the persistence of climate effects on fertility.

### 3.6. Data Standardization and Visualization

Because temperature and fertility have different scales and units, we standardize both series into z-scores to facilitate comparison across regions with differing baselines and variances. We calculate rolling five-year means to reduce interannual noise and highlight long-term trends. In our figures, we display both linear trends with 95% confidence bands and smoothed LOESS fits to visualize curvilinear components in the temperature–fertility association.

## 4. Results and Discussion

Before presenting the results, it is important to clarify the interpretation of the global temperature–fertility correlation. The reported global association is descriptively strong; however, both temperature anomalies and fertility rates exhibit pronounced secular trends over the 1960–2023 period. As such, part of this correlation likely reflects parallel long-run temporal dynamics rather than a direct temperature effect. The global bivariate association should therefore be interpreted primarily as evidence of large-scale co-movement rather than causal inference. Although ordinary least squares (OLS) models are commonly used in long-run comparative analyses, they may be sensitive to non-stationarity and shared time trends. For this reason, the regression results are interpreted cautiously as conditional associations rather than causal estimates.

To reduce the risk of spurious correlation and to account for demographic transition processes, we incorporate GDP per capita as a control variable in regional regression models. GDP per capita is used as a composite proxy for socioeconomic development and adaptive capacity, capturing broad structural transformations including improvements in income, infrastructure, healthcare access, and institutional resilience. While GDP is an informative and widely used indicator, it does not fully capture the multidimensional nature of demographic transition, which is also shaped by female education, urbanization, access to contraception, gender norms, migration, and cultural factors. Residual confounding from unmeasured demographic, social, and environmental variables may therefore remain.

It should be noted that the persistence of statistically significant temperature associations in certain regions after the adjustment to GDP does not establish causality. Rather, it suggests regional heterogeneity in the temperature–fertility relationship that may reflect differences in adaptive capacity and broader dimensions of socioeconomic development. GDP per capita is widely used as a composite proxy for development and adaptive capacity in both demographic and climate-vulnerability research [16,17,18], as it captures structural transformation, institutional strength, and resource availability that influence resilience to environmental stress. However, GDP cannot fully represent the multidimensional processes of development, including education, gender equity, healthcare access, and social norms. The observed regional variation therefore represents a strength of the manuscript, as it moves beyond global descriptive patterns and highlights how climate–demographic associations vary across developmental settings. Consistent with this framework, we describe temperature as a conditional demographic stressor whose statistical association with fertility depends on structural and institutional context, rather than as a confirmed biological driver of fertility decline.

### 4.1. Global Trends in Temperature and Fertility (1960–2023)

**Figure 5:**
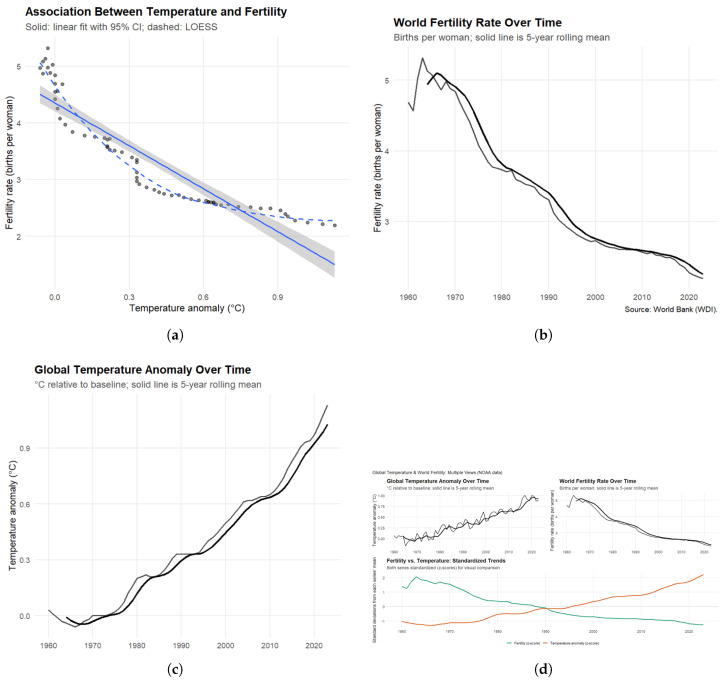
Association Between Global Temperature Anomalies and Fertility Rates (1960–2023). (**a**) Relationship between mean annual temperature anomalies (°C relative to baseline) and global fertility rates (births per woman). The solid line represents the linear regression fit with 95% confidence intervals (shaded area), while the dashed line shows a nonparametric LOESS fit. (**b**) Global fertility trends (births per woman) from 1960 to 2023, with a 5-year rolling mean; data source: World Bank (World Development Indicators). (**c**) Global surface temperature anomalies (°C relative to the 1951–1980 baseline) with a 5-year rolling mean; data source: NASA and NOAA. (**d**) Combined trends in temperature and fertility, standardized into *z*-scores for visual comparison. Together, the panels illustrate a strong and consistent negative association between global warming and fertility, robust across datasets and modeling approaches.

Figure 5 shows a strong negative association between global warming and reproductive outcomes. Mean global temperature increased by approximately 1 °C, while fertility rates fell by roughly half, from nearly five births per woman in the 1960s to just over two in recent years. We find a very strong correlation between temperature and fertility (r≈−0.9), explaining more than 80% of the variation in global fertility rates. Our lagged analyses confirm that this relationship persists across multiple years, indicating that climate effects on reproduction are lasting rather than short-term. Robustness checks using both NASA and NOAA datasets produce nearly identical results.

Rising temperatures and global warming may affect fertility through both direct physiological and indirect socioeconomic mechanisms. Physiologically, heat stress can impair sperm quality, disrupt ovulation, and increase pregnancy complications, thereby reducing reproductive success. Indirectly, climate-related economic strain, food insecurity, and migration may delay or limit family formation. The parallel timing of global fertility declines and accelerating anthropogenic warming suggests that climate change reinforces the broader demographic transition alongside education, urbanization, and economic development. If this pattern continues, it could alter population projections, labor force dynamics, and sustainability planning in the Anthropocene.

### 4.2. Global and Regional Regression Results

After accounting for major socioeconomic confounders, we report the regional associations between ambient temperature anomalies and fertility. These estimates describe how temperature–fertility correlations vary across regions with different levels of economic development and adaptive capacity, rather than implying a uniform or causal effect of climate on reproduction.

### 4.3. Addressing Confounding, Demographic Transition, and Adaptive Capacity

Population-level fertility decline is overwhelmingly shaped by socioeconomic development. A large literature documents that fertility falls with rising income, urbanization, female education, access to contraception, delayed marriage, and increasing costs of childrearing [12,13,14]. Desired family size responds strongly to improvements in child survival and women’s educational attainment, and the most effective fertility-reduction strategies combine human development with access to voluntary family planning [13,16]. These processes have unfolded across highly diverse climates and regions, from tropical South Asia to temperate Europe and highland Latin America, and account for most of the global fertility decline observed over the past six decades [64,71,72].

This study does not challenge the central role of socioeconomic development in fertility change. Rather, it evaluates whether rising ambient temperatures operate as a secondary constraint that interacts with economic and institutional conditions. Recent biomedical evidence demonstrates that heat exposure causally impairs both male and female reproductive function [6,15]. These biological sensitivities exist within social and economic environments that differ sharply in their capacity to buffer thermal stress through infrastructure, healthcare, occupational protections, and climate control technologies. Fertility decisions and reproductive outcomes therefore occur in contexts where heat exposure and adaptive capacity jointly shape demographic patterns.

Table 2 reports region-specific regressions that explicitly account for this confounding structure. Model (1) estimates the bivariate association between temperature anomalies and fertility. Model (2) adds log GDP per capita as a proxy for economic development and population-level adaptive capacity. GDP captures multiple components of the demographic transition, including urbanization, education, healthcare access, and institutional infrastructure, all of which mediate biological exposure to heat.

The results reveal strong regional heterogeneity. In Africa and the Middle East, temperature anomalies remain large and statistically significant predictors of fertility decline even after controlling for GDP. These regions are characterized by limited access to air conditioning, high levels of outdoor labor, weaker health systems, and constrained institutional capacity to buffer environmental stress. Under such conditions, thermoregulatory effects on spermatogenesis, pregnancy, and general health are more likely to translate into measurable demographic consequences.

In contrast, in South Asia, East Asia, Europe, and the Arctic, temperature effects become statistically insignificant once GDP is included. This pattern indicates that fertility decline in these regions is primarily governed by socioeconomic modernization, consistent with demographic transition theory. Higher adaptive capacity, including widespread climate control, robust healthcare systems, and regulatory protections, appears to mediate the biological sensitivity of reproduction to heat exposure.

The combined models explain a large share of fertility variation across regions, underscoring the dominance of economic and institutional forces in shaping reproductive behavior. The persistence of temperature effects only in regions with limited adaptive capacity suggests that climate warming acts as a conditional demographic stressor rather than a universal driver of fertility decline.

### 4.4. Asia Temperature Anomalies and Fertility Rates (1960–2023)

**Figure 6:**
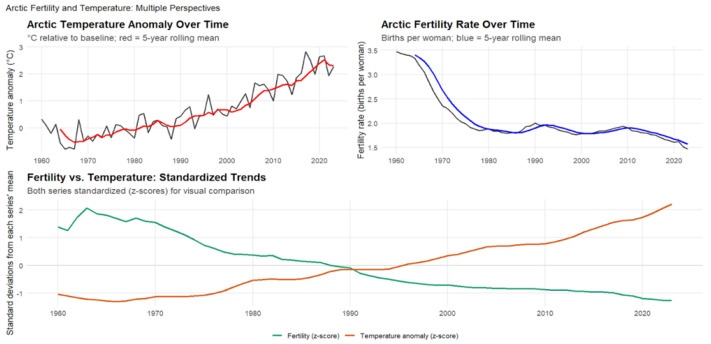
Arctic Temperature Anomalies and Fertility Rates, 1960–2023. This figure shows the relationship between temperature anomalies and fertility in nine Arctic nations. The **top-left** panel presents Arctic temperature anomalies relative to the long-term baseline, with the red line indicating the 5-year rolling mean. The **top-right** panel shows fertility rates (births per woman) with the blue line representing the 5-year rolling mean. The **lower** panel displays standardized (z-score) trends for both series to enable direct visual comparison.

**Figure 7:**
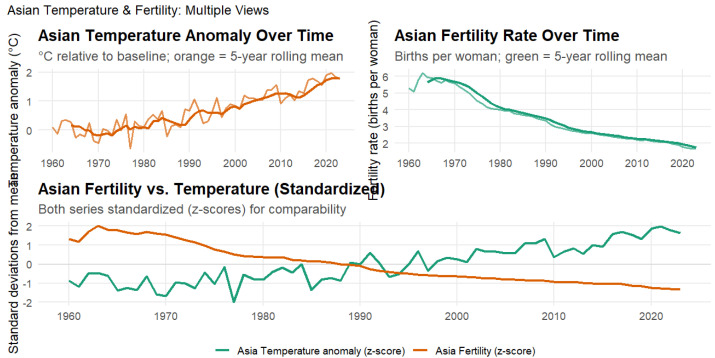
Asian Temperature Anomalies and Fertility Rates, 1960–2023. This figure illustrates the relationship between temperature anomalies and fertility across Asian nations. The **top-left** panel shows Asian temperature anomalies relative to the long-term baseline, with the orange line representing the 5-year rolling mean. The **top-right** panel presents fertility rates (births per woman), with the green line indicating the 5-year rolling mean. The **lower** panel displays standardized (z-score) trends for both variables to allow direct comparison.

Figure 6 and Figure 7 presents the relationship between temperature anomalies and fertility rates across Asia from 1960 to 2023. The analysis reveals a very strong negative correlation between regional warming and reproductive outcomes ($r=-0.83,\;p<2.2\times 10^{-16},\;95\%\;\mathrm{CI}:\;-0.90\;\mathrm{to}\;-0.74$), explaining approximately 70% of the variance in fertility rates. This represents one of the strongest regional climate–fertility associations observed globally. The relationship remained robust over time, with lagged correlations persisting for up to three years (r=−0.82,−0.81,and−0.80 for 1-, 2-, and 3-year lags, respectively), indicating that warming exerts prolonged rather than short-term effects on reproductive outcomes. The steady rise in temperature anomalies over six decades closely paralleled a sustained regional fertility decline, suggesting that climate change and demographic transition are progressing in tandem across Asia’s diverse economies.

The strength and persistence of this association reflect the interaction of climate warming with ongoing demographic transition across Asia’s diverse economies. While fertility decline in the region is primarily driven by urbanization, education, and changing family norms, sustained heat exposure may act as a secondary stressor that operates through health, labor productivity, and reproductive physiology. Variation in adaptive capacity across Asian countries likely mediates these effects, with populations lacking climate-controlled housing, occupational heat protection, or access to health care more exposed to environmental stress. These dynamics suggest that climate change and socioeconomic modernization are unfolding together, jointly shaping fertility trajectories across the world’s most populous continent.

### 4.5. African Temperature Anomalies and Fertility Rates (1960–2023)

**Figure 8:**
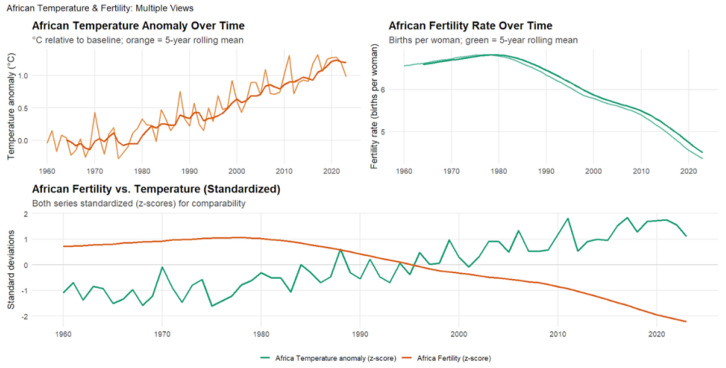
The figure shows a strong negative correlation between regional warming and fertility decline across Africa. The **upper** panels depict African temperature anomalies (°C relative to baseline; orange line with 5-year rolling mean) and fertility rates (births per woman; green line with 5-year rolling mean) from 1960 to 2023. The **lower** panel presents standardized (*z*-score) trends, illustrating how rising temperatures correspond closely with declining fertility over time.

Figure 8 illustrates patterns consistent with those observed in other regions, showing the strongest negative correlation of all regional analyses from 1960 to 2023 (r=−0.89, $p<2.2\times 10^{-16},\;95\%\;\mathrm{CI}:\;-0.94\;\mathrm{to}\;-0.83$). This relationship explains roughly 80% of the variance in fertility rates, indicating an exceptionally strong coupling between climatic and demographic change. Across the 64-year period, correlations remained highly stable and even intensified slightly over time, peaking at the two-year lag ($r=-0.91^{*}$), suggesting that climatic impacts on fertility operate through multi-year processes rather than immediate effects. Africa’s rapid warming, coupled with pronounced fertility decline across several subregions, highlights the deep interconnection between environmental stress and demographic transition.

The exceptional strength of the climate-fertility relationship in Africa reflects both structural vulnerabilities and broader dynamics shared across the Global South. Many African societies remain heavily dependent on agriculture and other climate-sensitive livelihoods, where extreme heat, droughts, and erratic rainfall directly threaten economic stability, health, and family formation decisions. These populations face compounding disadvantages: limited adaptive capacity, inadequate healthcare and infrastructure, and restricted access to reproductive health services amplify climate impacts. Climate shocks often trigger cascading consequences, migration, food insecurity, and social disruption, that extend well beyond the initial event, leading to long-term demographic changes. As Africa is projected to drive more than half of global population growth by mid-century, these findings suggest that climate change could accelerate fertility transitions and reshape population structures across the continent. More broadly, they highlight how climate pressures may intensify existing inequalities between the Global North and South, making climate adaptation not only an environmental imperative but also a demographic and social one. Integrating reproductive health, gender equity, and climate resilience into development planning will be essential for ensuring sustainable population outcomes in a rapidly warming world.

### 4.6. European Temperature Anomalies and Fertility Rates (1960–2023)

**Figure 9:**
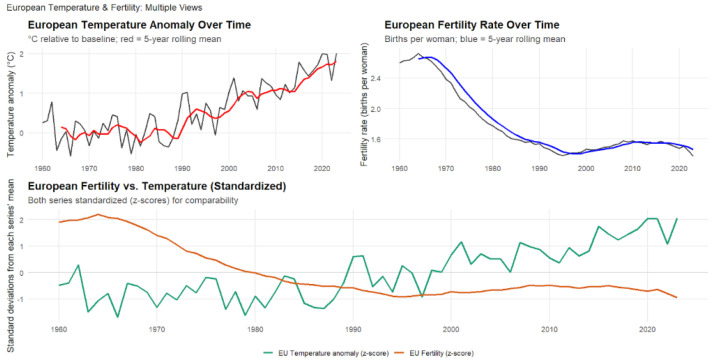
European Temperature Anomalies and Fertility Rates, 1960–2023. The red (temperature) and blue (fertility) lines represent centered 5-year rolling means, calculated to smooth short-term fluctuations and emphasize long-term trends. The **lower** panel presents standardized (z-score) values to facilitate direct comparison of relative changes over time.

As shown in Figure 9, European temperature anomalies and fertility rates from 1960 to 2023 exhibit a statistically significant negative correlation between regional warming and reproductive outcomes (r=−0.51,p<0.001,95%CI:−0.67to−0.31). Over this 64-year period, European temperatures increased by approximately 1.9 °C (0.03 °C per year), while fertility rates declined from 2.6 to 1.4 births per woman—a reduction of roughly 1.2 children per woman. The steepest fertility declines occurred between the 1960s and 1980s, after which rates stabilized at sub-replacement levels despite continued warming. Lagged correlations remained consistently negative across one- to three-year intervals (r=−0.49to−0.44), indicating that climate effects on fertility persist across multiple years even in highly developed populations. However, this correlation is substantially weaker than that observed in African countries (r=−0.9), likely reflecting Europe’s higher living standards, superior healthcare infrastructure, advanced social safety nets, and greater adaptive capacity to environmental stressors.

The European case provides important insight into how climate–demographic relationships operate in post-transitional societies. While Europe’s demographic transition was largely driven by socioeconomic development, expanding education, and increasing gender equality rather than direct climatic pressures, ongoing warming may be contributing to the persistence of low fertility and limiting recovery toward replacement levels. Several mechanisms could explain this relationship: physiological effects of heat stress on reproductive health, economic pressures associated with rising energy and adaptation costs, and heightened eco-anxiety influencing reproductive decisions and perceptions of future family stability. Unlike many populations in the Global South, Europe’s robust social welfare systems and healthcare infrastructure appear to mitigate the direct demographic consequences of climate change. Nevertheless, the persistent negative association suggests that even advanced economies are not immune to environmental influences on reproduction. As sub-replacement fertility converges with accelerated population aging, policymakers must increasingly consider how climate adaptation, immigration policy, and social welfare planning intersect with long-term demographic sustainability in an era of intensifying climate change.

### 4.7. Strengths, Limitations, and Policy Implications

This study has several important strengths. It provides comprehensive geographic and temporal coverage across 195 countries over 64 years (1960–2023), offering broad comparative evidence on climate–fertility co-movement across diverse socioeconomic and climatic contexts. The use of validated global datasets from the World Bank, NOAA, and NASA enhances transparency and reproducibility. Regional stratification with explicit adjustment for GDP per capita—used as a proxy for adaptive capacity—reveals heterogeneity that would be obscured in global descriptive analyses. Notably, temperature–fertility associations persist primarily in regions with more limited adaptive capacity (e.g., parts of Africa and the Middle East) and attenuate in higher-income regions, consistent with the interpretation of climate warming as a conditional demographic stressor. The analysis is informed by established thermophysiological literature on spermatogenesis and heat-sensitive reproductive pathways (e.g., CatSper, TRPV4, oxidative stress mechanisms), which provide biologically plausible context; however, these mechanisms are presented as supportive background rather than direct explanations of the demographic patterns observed.

Critical limitations constrain interpretation. The analysis relies entirely on country-level aggregate data and is therefore subject to ecological fallacy. Associations observed at the population level cannot be interpreted as evidence of individual-level reproductive impairment or as confirmation of specific biological mechanisms. National fertility rates reflect complex demographic processes shaped by education, urbanization, gender norms, contraceptive access, economic structure, and health systems, and cannot be directly mapped onto sperm parameters or physiological endpoints. Accordingly, the biological mechanisms discussed should be understood as plausible pathways consistent with experimental literature, not as causal inferences drawn from ecological data.

In addition, the very strong global correlation between temperature anomalies and fertility decline (r≈−0.90) must be interpreted cautiously. Both variables exhibit pronounced secular trends over time, and part of this association likely reflects parallel long-run global trajectories rather than direct temperature effects. These global correlations are therefore primarily descriptive indicators of co-movement. Although regional models adjusting for GDP per capita reduce this concern, residual confounding remains possible. GDP does not fully capture the multidimensional nature of demographic transition, including female education, urbanization, contraceptive access, migration, environmental pollution, nutrition, and infectious disease burden. The pathways linking ambient temperature to fertility are likely multifactorial—potentially involving physiological stress, behavioral responses, institutional capacity, and environmental co-exposures—and cannot be disentangled using country-level ecological data. Rigorous individual-level longitudinal studies with detailed exposure assessment and biological endpoints are necessary to evaluate the causal pathways suggested by these patterns.

Across global and regional analyses, strong and statistically significant negative correlations were observed between temperature anomalies and fertility, with the largest associations in Africa (r=−0.89), Asia (r=−0.83), and the Arctic (r=−0.55) (See Figure 6). From a policy perspective, these findings underscore the importance of integrating climate adaptation planning with reproductive and public health systems, particularly in regions with limited adaptive capacity. Even if climate warming operates as a conditional or amplifying stressor rather than a primary driver of demographic change, strengthening heat resilience—through occupational protections, cooling infrastructure, healthcare system preparedness, and environmental pollution mitigation—may reduce vulnerability to climate-related reproductive risks. Future interdisciplinary research linking climate science, reproductive biology, demography, and public health will be essential for translating population-level correlations into evidence-based adaptation strategies.

## 5. Conclusions

Fertility decline over the past six decades has been driven primarily by socioeconomic modernization, including rising income, female education, urbanization, and access to contraception. These forces explain most of the global reduction in births per woman. Our analyses identify a consistent secondary associative pattern in which rising ambient temperatures correlate with lower fertility, particularly in regions with limited adaptive capacity.

These are associative patterns and do not establish causation. The observed temperature–fertility relationships could reflect unmeasured confounding from factors that co-vary with both temperature and fertility, including air pollution, water quality, infectious disease burden, agricultural productivity, migration patterns, and conflict. The pathways linking ambient temperature to population-level fertility remain uncertain and are likely multifactorial, involving direct physiological mechanisms, indirect behavioral responses, health system impacts, or synergistic interactions with other environmental stressors.

When economic development is accounted for, temperature associations become statistically insignificant in high-income regions but persist in lower-income settings, suggesting that adaptive capacity effectively buffers environmental stress where available. However, distinguishing between direct physiological effects and indirect socioeconomic pathways requires individual-level longitudinal studies with detailed exposure assessment and rigorous control for behavioral and environmental confounders.

Future research should link personal heat exposure to reproductive outcomes through individual-level and subnational data. Integrating physiological temperature thresholds into demographic and climate projection models would improve population change forecasts under continued warming.

These findings suggest that climate warming may interact with demographic transition in settings where adaptive capacity is limited. As global warming continues, differences in adaptive capacity may increasingly shape the geography of population change, making climate adaptation a central component of long-term demographic stability.

## Figures and Tables

**Table 2:** Temperature, Economic Development, and Fertility Across Regions.

	Afr(1)	Afr(2)	S.As(1)	S.As(2)	E.As(1)	E.As(2)	M.E(1)	M.E(2)	Eur(1)	Eur(2)	Arc(1)	Arc(2)
Intercept	6.70 ***	11.54 ***	5.56 ***	29.25 ***	3.87 ***	21.39 ***	6.37 ***	18.50 ***	1.96 ***	12.50 ***	2.25 ***	16.72 ***
	(0.06)	(1.65)	(0.12)	(1.46)	(0.15)	(2.72)	(0.14)	(1.93)	(0.06)	(0.51)	(0.07)	(1.35)
Temperature Anomaly	−1.45 ***	−0.99 ***	−1.94 ***	−0.14	−1.57 ***	0.10	−2.29 ***	−1.27 ***	−0.32 ***	0.16 ***	−0.29 ***	0.26 ***
	(0.09)	(0.18)	(0.12)	(0.12)	(0.17)	(0.29)	(0.15)	(0.20)	(0.07)	(0.03)	(0.06)	(0.06)
Log GDP		−0.19 **		−0.99 ***		−0.70 ***		−0.52 ***		−0.37 ***		−0.54 ***
		(0.07)		(0.06)		(0.11)		(0.08)		(0.02)		(0.05)
$R^{2}$	0.80	0.82	0.80	0.96	0.59	0.76	0.80	0.88	0.26	0.91	0.30	0.76
Adjusted $R^{2}$	0.80	0.82	0.79	0.96	0.59	0.75	0.80	0.87	0.25	0.90	0.29	0.75
Observations	64	64	64	64	64	64	64	64	64	64	64	64

Notes: OLS regressions using annual data (1960–2023). Models (1) include temperature anomaly only; models (2) additionally control for log GDP. Robust standard errors in parentheses. *** p<0.001, ** p<0.01.

## Data Availability

The data presented in this study are available on request from the corresponding author.

## Abbreviations

The following abbreviations are used in this manuscript:

TFR	Total Fertility Rate
NOAA	National Oceanic and Atmospheric Administration
NASA	National Aeronautics and Space Administration
IPCC	Intergovernmental Panel on Climate Change
ROS	Reactive Oxygen Species
ATP	Adenosine Triphosphate
TSGA10	Testis-Specific Gene 10
CytC1	Cytochrome c1
COX	Cytochrome c Oxidase
ERSST	Extended Reconstructed Sea Surface Temperature
GHCN-M	Global Historical Climatology Network-Monthly
LOESS	Locally Estimated Scatterplot Smoothing
OLS	Ordinary Least Squares
UHI	Urban Heat Island
UN DESA	United Nations Department of Economic and Social Affairs
SDG	Sustainable Development Goals
CI	Confidence Interval
